# Renal replacement therapy in adult and pediatric intensive care: Recommendations by an expert panel from the French Intensive Care Society (SRLF) with the French Society of Anesthesia Intensive Care (SFAR) French Group for Pediatric Intensive Care Emergencies (GFRUP) the French Dialysis Society (SFD)

**DOI:** 10.1186/s13613-015-0093-5

**Published:** 2015-12-30

**Authors:** Christophe Vinsonneau, Emma Allain-Launay, Clarisse Blayau, Michael Darmon, Damien du Cheyron, Theophile Gaillot, Patrick M. Honore, Etienne Javouhey, Thierry Krummel, Annie Lahoche, Serge Letacon, Matthieu Legrand, Mehran Monchi, Christophe Ridel, René Robert, Frederique Schortgen, Bertrand Souweine, Patrick Vaillant, Lionel Velly, David Osman, Ly Van Vong

**Affiliations:** Réanimation polyvalente, CH Melun, 77000 Melun, France; CHU Nantes, Nantes, France; CHU Tenon, 75020 Paris, France; CHU Saint-Etienne, 42055 Saint-Etienne, France; CHU Caen, 14033 Caen, France; CHU Rennes, 35000 Rennes, France; Intensive Care Department, Universitair Ziekenhuis Brussel, Vrije Universiteit Brussel, Brussels, Belgium; Réanimation pédiatrique spécialisée, CHU Lyon, 69677 Bron, France; CHRU Strasbourg, Strasbourg, France; CHRU Lille, Lille, France; CHU Nancy, Nancy, France; CHU Saint-Louis Lariboisière, Paris, France; CHU Poitiers, Poitiers, France; CHU Mondor, 94000 Créteil, France; CHU Clermont-Ferrand, Clermont-Ferrand, France; CHU Necker, 75000 Paris, France; CHU Marseille, Marseille, France; CHU Bicêtre, 94, Le Kremlin Bicêtre, France

**Keywords:** Renal replacement therapy, Anticoagulation, Dialysis dose, Continuous renal replacement therapy, Hemodialysis, Citrate, Recommendations

## Abstract

Acute renal failure (ARF) in critically ill patients is currently very frequent and requires renal replacement therapy (RRT) in many patients. During the last 15 years, several studies have considered important issues regarding the use of RRT in ARF, like the time to initiate the therapy, the dialysis dose, the types of catheter, the choice of technique, and anticoagulation. However, despite an abundant literature, conflicting results do not provide evidence on RRT implementation. We present herein recommendations for the use of RRT in adult and pediatric intensive care developed with the Grading of Recommendations Assessment, Development, and Evaluation (GRADE) system by an expert group of French Intensive Care Society (SRLF), with the participation of the French Society of Anesthesia and Intensive Care (SFAR), the French Group for Pediatric Intensive Care and Emergencies (GFRUP), and the French Dialysis Society (SFD). The recommendations cover 4 fields: criteria for RRT initiation, technical aspects (access routes, membranes, anticoagulation, reverse osmosis water), practical aspects (choice of the method, peritoneal dialysis, dialysis dose, adjustments), and safety (procedures and training, dialysis catheter management, extracorporeal circuit set-up). These recommendations have been designed on a practical point of view to provide guidance for intensivists in their daily practice.

## Background

The prevalence of acute renal failure (ARF) in intensive care, in an unselected population, is high—about 40 %—and a renal replacement therapy technique is required in just under 20 % of patients presenting ARF [1]. The use of consensual definitions (RIFLE, KDIGO) for the diagnosis and assessment of the severity of renal failure enables comparison of types of practice, such as the use of techniques of renal replacement therapy (RRT) [2]. There are large disparities between studies. The FINNAKI group reported that prevalence of RRT ranged from 3 to 36 % among intensive care units, whereas patient mortality did not differ [3]. The absence of consensual criteria for use of RRT generates great variability, notably in populations of septic patients for whom some teams use RRT in indications other than acute kidney failure. Numerous studies over the last 15 years have considered the time to initiation of RRT, dialysis dose, types of catheter, choice of technique, and anticoagulation. The last SRLF recommendations on continuous dialysis (1997) are now old, and the 2012 international recommendations on management of renal failure (ATS-ERRT-ESICM-SCCM-SRLF) only partially consider RRT. It therefore seemed necessary to draw up these recommendations for the practical aspects of RRT to provide guidance for intensivists in their daily practice. Most aspects of RRT in adult or pediatric ICU have been considered in these recommendations, but some of them have been unheeded in regard to the lack of data in the literature or the prioritization decided by the panel expert.

## Methodology

These recommendations were drawn up by a panel of experts brought together by the SRLF in collaboration with scientific societies in disciplines that contribute to the management of acute renal failure by RRT: French Society of Anesthesia and Intensive Care (SFAR), French Group for Pediatric Intensive Care and Emergencies (GFRUP) and the French Dialysis Society (SFD). The organizing committee appointed a coordinator who selected the SRLF experts. Each scientific society associated with these expert recommendations selected its experts. The coordinator first defined the questions to be covered and proposed experts to be in charge of each question. A first meeting was organized to discuss the proposed questions and the choice of experts to cover them, and to approve everything. Literature analysis and formulation of recommendations were then performed using the GRADE system (Grade of Recommendation Assessment, Development and Evaluation) [4, 5]. A level of proof was defined for each bibliographic reference as a function of the type of study. This level of proof could be reassessed taking into account the methodological quality of the study. The bibliographic references common to each outcome were then pooled. An overall level of evidence was determined for each outcome, taking into account the level of proof of each bibliographic reference, the consistency of the results between the different studies, the direct or indirect nature of the proof, cost analysis, etc. A “strong” level of proof enabled formulation of a “strong” recommendation (should be done, should not be done). A “moderate,” “weak,” or “very weak” level of proof led to the drawing up of an “optional” recommendation (should probably be done, should probably not be done …). The proposed recommendations were presented and discussed at a second meeting of all the experts. The aim was not necessarily to reach a single and convergent opinion of the experts regarding all proposals, but to bring out the points of agreement and the points of disagreement or of indecision. Each recommendation was then evaluated by each expert who rated it according to a scale from 1 (complete disagreement) to 9 (complete agreement). The collective score was established using a methodology derived from the RAND/UCLA appropriateness method [6]: after elimination of the extreme values (outliers), the median and confidence interval of the scores were calculated. The median defined disagreement between the experts when it was between 1 and 3, agreement between 7 and 9 and indecision between 4 and 6. The disagreement, agreement, or indecision was “strong” if the confidence interval was within one of three ranges: (1–3), (4–6) or (7–9) and “weak” if the confidence interval straddled two ranges. In the absence of strong agreement, the recommendations were reformulated and again scored with a view to achieving a better consensus. Two rounds of scoring were therefore performed.

## Area 1: Criteria for initiation of RRT in renal failure

1.1 RRT should be initiated without delay in life-threatening situations (hyperkalemia, metabolic acidosis, tumor lysis syndrome, refractory pulmonary edema). (Expert opinion) Strong agreement
1.2 The available data are insufficient to define optimal timing of initiation of RRT outside life-threatening situations. (Expert opinion) Strong agreement

Despite the lack of a specific study, the benefit of RRT in life-threatening situations seems reasonable, which explains why most experts recommend its use in such circumstances [7]. Is it useful to initiate it earlier in acute renal failure? Several observational, randomized studies and meta-analyses have examined the benefit of early initiation [8–23], but the findings are discordant. Three prospective randomized studies showed no benefit [9], a deleterious effect [11] or a net benefit [10]. Three prospective open studies found no benefit of early initiation of RRT [12–14]. Several observational retrospective studies of low methodological quality, mostly without adjustment for confounding factors, suggest a benefit [15–23]. A meta-analysis of these data suggests a benefit of early initiation of RRT [8]. The low level of proof of most studies considered, the diversity of definitions of early or late initiation, the heterogeneity of the study populations, unequal quality of available data, observed bias, including a strongly suspected publication bias, and the small number of patients in these studies mean that definitive conclusions cannot be drawn. The findings of the literature analysis are insufficient to enable a recommendation concerning the optimal timing of RRT initiation, outside life-threatening situations.

1.3 In children, fluid and sodium overload probably of above 10 %, and very probably of above 20 %, should be considered as one of the criteria for initiation of renal replacement therapy. (Expert opinion) Poor agreement

Several observational studies have shown that fluid overload before initiation of RRT is a mortality risk factor, including multivariate analysis [24–29]. All studies indicate that the threshold of 20 % fluid overload is associated with a large increase in mortality (odds ratio 8.3) [28]. Between 10 and 20 %, the increase in mortality is less clear. Foland et al. showed that a fluid overload of more than 10 % is enough to increase mortality, but only in the group of children with dysfunction of ≥ 3 organs [25]. While all these data suggest a strong association between mortality and fluid overload, no study has compared a strategy of intervention by RRT according to different fluid overload thresholds, such that the level of proof is low and no causal link can be established.

1.4 “Early” initiation of RRT means at KDIGO stage 2 or within 24 h after onset of acute renal failure of which reversibility seems unlikely. (Expert opinion) Poor agreement
1.5 “Late” initiation of RRT means over 48 h after onset of acute renal failure, KDIGO stage 3, or when a life-threatening situation arises because of acute renal failure. (Expert opinion) Poor agreement

Although the data are insufficient to recommend early initiation of RRT, it seems necessary to pursue research in this field [8]. In this regard, the control group should mimic usual prescribing habits as best as possible [30–33]. The early or late nature of the intervention should therefore be defined as modifying usual practice. The KDIGO classification [2], which is used to assess the severity of renal failure, seems to be the most suited to defining the intervention groups.

## Area 2: Technical aspects

### Area 2.1: Access routes

2.1.1 The subclavian route should be avoided. (Expert opinion) Strong agreement

On the basis of old data on end-stage renal failure patients on dialysis, the CDC (http://www.cdc.gov/dialysis/guidelines) and KDOQI (http://www.kidney.org/professionals/kdoqi/guidelines_commentaries.cfm) recommend avoiding the placement of temporary RRT catheters at the subclavian site because of a risk of stenosis of the subclavian and axillary veins, which could compromise the function of, or the future use of, a permanent access such as an arteriovenous fistula if acute renal failure progresses to end-stage renal disease [34]. Recent data show that progression to chronic renal failure after acute kidney injury occurs in close to 25 % of cases, when combining chronic dialysis and doubling of plasma creatinine [35]. In terms of the incidence rate, this risk is assessed as 7.8 events/100 patient-years for the presence of an acute kidney injury and 4.9 events/100 patient-years for the presence of end-stage renal disease [36]. In view of these data, it seems right not to use the subclavian route as the vascular access for RRT in renal failure.

2.1.2 The femoral vein and right internal jugular vein sites should be considered as equivalent in terms of infectious complications. Strong agreement
2.1.3 The internal jugular site should probably be used to reduce catheter-related infection risk for patients with a body mass index above 28 kg/m^2^. Strong agreement

An infectious complication is a local or general infection secondary to the presence of microorganisms at the internal and/or external surface of the catheter. The results of nonrandomized studies on the central venous catheter are divergent [37, 38]. The femoral route is reputed to be associated with an increased risk of colonization and infection compared with the jugular route. However, nonrandomized studies did not find an increased risk of bacteremia with central venous catheterization comparing femoral versus internal jugular vein insertion [39, 40]. Although the epidemiology of infections associated with dialysis catheters is similar to the epidemiology of infections associated with central venous catheters, the extrapolation of these results is difficult. There is a single randomized study in a small population comparing the femoral and internal jugular sites for RRT with colonization as the main outcome measure [41]. The femoral site did not significantly increase the risk of colonization of the first catheter (40.8 versus 35.7 for 1000 catheter-days for the jugular route; *P* = 0.31) and the rate of catheter-related bloodstream infection was similar (1.5 versus 2.3 for 1000 catheter-days for the jugular route; *P* = 0.42). Only the subgroup of patients with a body mass index above 28 kg/m^2^ had an increased infectious risk associated with the femoral site. In the same prospective randomized study of RRT, the absence of difference in colonization between the jugular and femoral sites was confirmed by a cross-over analysis of the second catheterization [42].

2.1.4 The femoral and right internal jugular sites should be considered as equivalent in terms of the risk of catheter dysfunction. Poor agreement

A single prospective randomized study has compared the internal jugular and femoral sites in terms of catheter dysfunction and efficacy of RRT [43]. Catheter dysfunction was similar at the internal jugular (11.1 %) and femoral (10.3 %) sites. The efficacy of RRT, arbitrarily defined by the urea reduction rate in intermittent hemodialysis and by the continuous RRT downtime, was comparable for the internal jugular and femoral sites. In contrast, compared with the jugular site, the femoral site was subject to more dysfunction and blood recirculation when the catheter length was <25 cm or if the blood flow was >200 mL/min, as reported in patients treated by intermittent hemodialysis [43, 44]. The femoral and jugular sites can therefore be considered equivalent in terms of catheter dysfunction, provided use is made of a catheter appropriate to the femoral route.

2.1.5 The left internal jugular site should probably be kept as a third choice. Strong agreement

A single study has directly compared the rate of catheter dysfunction according to right or left internal jugular placement, with respect to femoral placement [43]. In this subgroup analysis, the risk of catheter dysfunction was significantly higher for the left internal jugular site than for the femoral site or the right internal jugular site.

2.1.6 In children, right internal jugular access should probably be preferred to femoral access for children weighting less than 20 kg (or if the catheter is <10F). Poor agreement
2.1.7 Catheter size should be adapted to the child’s morphology and body weight. (Expert opinion) Strong agreement
  3–6 kg → 6.5–7 F
  6–10 kg → 8 F
  10–20 kg → 8–10 F
  20–30 kg → 10 F
  >30 kg → 11–13 F

In children, the preferred insertion site is the right internal jugular vein in terms of the quality of dialysis [45] and optimization of hemofilter life [46], in particular for small-caliber catheters (<10 F) [47]. The choice of this route should take into account the usual precautions concerning the inherent risk of a “high” placement, notably in cases of respiratory distress or coagulopathy. The femoral site is an acceptable alternative, and is feasible whatever the patient’s condition. The caliber of the catheter should be adapted to the child’s body weight, favoring catheters of relatively large caliber [47, 48].

2.1.8 For the femoral site, choose catheters of diameter >12 F and length ≥24 cm. (Expert opinion) Strong agreement

Blood flow through the catheter is one of the major determinants of the quality of dialysis [49]. The degree of recirculation should also be taken into account, even though it is generally low (<10 %) when a double-lumen catheter is used for vascular access. This percentage, which corresponds to the proportion of recirculated blood in the catheter, increases when there is turbulence in the vein related to low blood flow, in the case of partial thrombosis of the catheter and/or of the vein at catheter insertion, and/or if the routes are reversed. To ensure good catheter performance and effective dialysis, the distal end should be positioned in a large-caliber vein with a high blood flow, so that the quantity of blood available in the vessel is never limiting. In patients with end-stage renal failure, temporary dialysis catheters shorter than 20 cm generate more recirculation than catheters over 20 cm in length [50]. These results were confirmed in intensive care in a post hoc analysis of the Cathedia study where femoral catheters below 24 cm in length were associated with a decrease in the urea reduction rate compared with catheters longer than 24 cm [43].

2.1.9 Ultrasound guidance should be used for catheter placement in the internal jugular vein in RRT. Strong agreement

Ultrasound guidance, in accordance with current international recommendations, is considered as the reference technique for placement of central venous catheters in intensive care. By guiding insertion in the vein and detecting anatomical variations, ultrasound improves the success rate and patient comfort, and reduces the time needed for placement, the number of attempts per insertion, the number of complications and cost [51–53]. Few studies have specifically considered dialysis catheters in intensive care. A 2011 meta-analysis of 7 randomized studies (830 catheters in total; 3 studies reported only as abstracts) compared the placement of 380 dialysis catheters based solely on anatomical landmarks with ultrasound-guided placement of 450 dialysis catheters [54]. Most of the catheters (85 %) studied were inserted in the internal jugular vein. Ultrasound guidance was associated with a reduced risk of catheter placement failure compared with placement using anatomical landmarks (RR = 0.12; 95 % CI 0.04–0.37). Likewise, ultrasound guidance reduced catheter placement failure at the first attempt (RR = 0.4; 95 % CI 0.29–0.56), the number of attempts, the time taken to cannulate the vein, as well as the number of complications such as arterial puncture, hematoma, and pneumothorax [55–57].

2.1.10 Ultrasound guidance should probably be used for femoral vein catheter placement in RRT. Poor agreement

One randomized single-center study specifically compared the placement of dialysis catheters by the femoral route using ultrasound guidance or anatomical landmarks [58]. The success rate for placement of 110 double-lumen dialysis catheters was 98.2 % with ultrasound guidance and 80 % using anatomical landmarks (p = 0.002; odds ratio = 13.5, 95 % CI 1.7–108.7). The success rate at the first attempt was clearly higher with ultrasound guidance (85.5 versus 54.5 %, p = 0.000). The rate of complications decreased from 18.2 to 5.5 % (p = 0.039) with ultrasound guidance. This study therefore shows that ultrasound guidance of femoral dialysis catheter placement reduces the failure rate, the number of attempts at femoral venous puncture and the incidence of complications related to catheter placement, in accordance with all data and recommendations concerning the placement of central venous catheters. However, the paucity of data and the small patient numbers prevent a strong recommendation.

2.1.11 Catheters for RRT should be removed as soon as they are no longer necessary. (Expert opinion) Strong agreement

Temporary dialysis catheters should be considered as a central venous line at risk of infection and mechanical complications and a potential source of increased morbidity and mortality. Their usefulness should therefore be reassessed daily and they should be removed once RRT is no longer necessary.

2.1.12 An arteriovenous fistula should not be used in the absence of relevant expertise. (Expert opinion) Strong agreement

An arteriovenous fistula is an anastomosis between an artery and a superficial vein, generally situated on the forearm or inner face of the arm, which gives reliable and functional vascular access for dialysis of patients in chronic renal failure. Given the potential complications associated with repeated puncture of the arteriovenous fistula (skin damage, recirculation, aneurysm, local or systemic infection, thrombosis and hematoma), everything should be done to preserve this vascular access [59]. Thus, the puncture of an arteriovenous fistula by a physician or nurse calls for particular expertise. If this expertise is lacking, the vascular access for RRT requires the placement of a double-lumen catheter in the internal jugular vein or femoral vein.

### Area 2.2: Membranes

2.2.1 Unmodified cellulose membranes (Cuprophan) should probably not be used for the management of patients with acute renal failure. Strong agreement

The biocompatibility of dialysis membranes is generally defined by their capacity to activate mediators of inflammation (essentially the alternative complement pathway) and granulocytes, in vitro. However, in clinical use, the activation of inflammation at the membrane does not only depend on the membrane characteristics, but also on anticoagulation and on the quality of the dialysis (or replacement) fluid. Biocompatibility therefore depends on the whole dialysis system (type of membrane, anticoagulation, fluid used). Randomized studies of the survival of acute renal failure patients in intensive care according to the type of membrane used [60–75] do not yield a definitive conclusion because of the small total number of patients studied (under 650). However, meta-analyses suggest excess mortality with Cuprophan membranes, at the limit of significance. It therefore seems prudent to avoid the use of Cuprophan membranes in intensive care. In contrast, the use of cellulose acetate membranes does not seem to be associated with a risk of excess mortality. There is no trend to superiority of a given type of synthetic membrane over another.

2.2.2 Use should be made of membranes of high hydraulic permeability (high ultrafiltration coefficient) for convective dialysis techniques (hemofiltration). (Expert opinion) Strong agreement
2.2.3 In intermittent hemodialysis, membranes of high hydraulic permeability should not be used in the absence of ultrapure dialysate. (Expert opinion) Strong agreement

The hydraulic permeability of a membrane is defined by an ultrafiltration coefficient (in mL/h/mmHg/m^2^ of membrane area). This permeability is proportional to the number of pores per unit area and to the mean radius of the pores to the power 4. Convective dialysis techniques (hemofiltration) require a high hydraulic permeability (≥20 mL/mmHg/m^2^/h), to permit a sufficient ultrafiltration flow rate without excessive pressure. Diffusive methods (hemodialysis) are, on the other hand, perfectly compatible with a low ultrafiltration coefficient (<5 mL/mmHg/m^2^/h). The use of a membrane of high ultrafiltration coefficient in hemodialysis can generate filtration-backfiltration movements during treatment which may provoke the passage of endotoxin or microbial debris.

2.2.4 It does not seem useful to use a membrane of high porosity (high cutoff) or of high adsorption capacity for the treatment of septic shock. (Expert opinion) Strong agreement

One of the characteristics of an RRT membrane is its permeability limit or cutoff. Below the cutoff, in hemofiltration, each molecule has a sieving coefficient, defined by the ratio of [concentration of the molecule in the ultrafiltrate]/[concentration of the molecule in the plasma]. This ratio is 1 for small molecules and generally <0.1 for molecules of molecular weight above 15 kDa. Membranes with a high hydraulic permeability also generally have a high permeability to medium-sized molecules (porosity). There is no evidence of a significant difference in survival rate with membranes of high permeability or high porosity in acute renal failure patients. The same is true of so-called superpermeable membranes or membranes of very high porosity, with in vitro cutoffs of 60–100 kDa (≤60 kDa in vivo). These membranes were created to improve removal of medium-sized molecules, immunoglobulin light chains and cytokines, at the cost, however, of more or less large loss of albumin. There is no comparative study of mortality in intensive care patients with these superpermeable membranes. The studies are preliminary and the value of removing cytokines in septic shock is not established. Likewise, there is no study comparing the survival of patients according to the adsorption capacity of the membranes used. No specific membrane can, therefore, be recommended in the management of septic shock patients.

2.2.5 Heparin-coated or heparin-binding membranes should probably not be used to reduce anticoagulation of the RRT circuit. Strong agreement

Certain specifically treated membranes bind 5 times more heparin on rinsing than other membranes. Others are coated with heparin. The value of these membranes in RRT in intensive care patients has not been demonstrated. Studies of their use to avoid systemic anticoagulation are contradictory and so no definitive conclusion can be drawn [76–79].

### Area 2.3: Anticoagulation

2.3.1 In patients at high risk of hemorrhage or presenting coagulopathy:
2.3.1.1 In intermittent RRT, systemic anticoagulation should probably be avoided. Poor agreement
2.3.1.2 In continuous RRT, regional citrate anticoagulation should probably be preferred to no anticoagulation, unless there is a contraindication. Strong agreement
2.3.1.3 In continuous RRT, no anticoagulation should probably be preferred if there is a contraindication to citrate. (Expert opinion) Poor agreement

In patients at high risk of hemorrhage, the use of systemic anticoagulation is not recommended when RRT is indicated, as the expected advantage regarding the circuit is outweighed by the risk of bleeding.

The use of regional citrate anticoagulation seems to be the method of choice in these situations. In intermittent hemodialysis, however, the lack of a secure administration procedure makes its use complex.

In continuous dialysis, the strictly regional nature (only the circuit) of citrate anticoagulation means that it is preferred for first-line use. Three observational studies [80–82] in patients at high risk of hemorrhage have reported a significant decrease in hemorrhagic complications with citrate use. For ethical reasons, no randomized trial has compared citrate with heparin when there is a high risk of hemorrhage. However, most prospective randomized studies [83–87] have shown a significant reduction in hemorrhagic complications in the citrate group, despite the low risk of hemorrhage of the study populations.

When citrate is contraindicated, no anticoagulation should be preferred to systemic heparin anticoagulation. However, nonuse of anticoagulation in RRT exposes the patient to high consumption of coagulation factors. No study has demonstrated the value of circuit adjustments designed to limit thrombosis, such as the use of a high predilution dose or periodic rinsing.

Regional heparin-protamine anticoagulation is not recommended, irrespective of the method of dialysis (continuous or intermittent). It exposes patients to the side effects of both heparin (notably the risk of heparin-induced thrombocytopenia) and protamine (principally anaphylaxis, platelet dysfunction, hypotension, and pulmonary vasoconstriction with a risk of right ventricular failure) [88]. As heparin has a much longer half-life than protamine, this technique is difficult to titrate and also exposes patients to a rebound effect upon withdrawal of treatment.

2.3.1.4 In children, continuous RRT can be done without anticoagulation or by using regional citrate anticoagulation, the choice being guided by the experience of the team. (Expert opinion) Strong agreement

The use of citrate in dialysis in children offers an interesting alternative to heparin, notably for patients at high risk of hemorrhage. Few pediatric studies have been conducted and none is sufficiently relevant to conclude that citrate is superior to heparin. The recommendations formulated are thus those of experts.

Bunchman et al. reported filter lifetimes of approximately 72 h on average, in a series of 14 patients on continuous venovenous hemofiltration with dialysis [89] and in another series of 9 patients on continuous venovenous hemofiltration [90], without hemorrhagic complications. In a study of 9 patients, Elhanan et al. reported filter survival of up to 5 days [91].

In comparison with heparin, the observational study of Brophy et al. [92] in a cohort of 138 patients (93 heparin, 37 citrate, 9 no anticoagulation) showed increased hemorrhagic complications with heparin, an identical filter lifespan (heparin versus citrate) and increased filter lifespan (citrate anticoagulation/heparin versus no anticoagulation).

As for toxicity/risk of citrate accumulation, Chadha et al. [93] showed, in a series of 5 patients undergoing continuous venovenous hemofiltration, satisfactory clearance of calcium-citrate complexes. The risks in terms of biochemical values (metabolic acidosis or alkalosis, hypocalcemia) should be known and closely monitored using point-of-care testing. Concentrated citrate increases the risk of citrate toxicity [93], whereas lower concentrations may increase effluent flow rate to above recommended values [94]. These risks must be taken into account when choosing the concentration of citrate solution, which varies between suppliers, whence the need for specialist medical expertise.

Lastly, an observational study of 344 patients [95], 57 % of whom were treated with citrate, shows that the technique is widely used by North American teams and probably underused by European teams.

These observational studies of small populations do not enable a recommendation to be made with a sufficient level of proof. The use of citrate by pediatric teams calls for great mastery of the prescription and of the biochemical monitoring.

2.3.2 In patients at low risk of hemorrhage not requiring systemic anticoagulation:
2.3.2.1 In intermittent RRT, unfractionated heparin or low-molecular-weight heparin should probably be preferred to other systemic anticoagulants. (Expert opinion) Strong agreement
2.3.2.2 In continuous RRT, in adults, regional citrate anticoagulation should probably be preferred, unless there is a contraindication, so as to prolong circuit lifetime. Poor agreement
2.3.2.3 In continuous RRT, if there is a contraindication to citrate, unfractionated heparin anticoagulation should probably be preferred. (Expert opinion) Strong agreement

In intermittent dialysis, a meta-analysis of 11 randomized studies in patients with end-stage renal failure found no difference between unfractionated heparin and low-molecular-weight heparin in terms of bleeding complications or antithrombotic efficacy [96]. If low-molecular-weight heparin is chosen, its dosage should be adapted (elimination principally renal) to prevent a risk of accumulation and the occurrence of hemorrhagic complications. The dose used in intermittent dialysis is below that used in therapeutic anticoagulation, notably in scheduling the injections because of the long half-life of low-molecular-weight heparin. Regular anti-Xa activity assay seems desirable when use is prolonged.

In continuous dialysis, regional citrate anticoagulation significantly extends circuit lifetime compared with heparin. This recommendation is based on the results of 6 randomized studies and 8 observational studies that compared regional citrate anticoagulation with heparin anticoagulation in patients requiring continuous RRT [80, 82–87, 92, 97–103]. Regional citrate anticoagulation also limits hemorrhagic complications [80–87, 98–100, 102, 103], economizes use of blood [80, 82, 83, 85–87, 101] and limits consumption of platelets [87, 98–100, 102], without altering mortality [80, 87, 97, 98, 100, 104].

If citrate anticoagulation is contraindicated or unavailable, unfractionated heparin (partial thromboplastin time target 1.5 times the control) was preferred by the experts because of its pharmacokinetics and its reversibility compared with low-molecular-weight heparin.

2.3.2.4 In children, in continuous RRT, citrate or unfractionated heparin should be used for anticoagulation, the choice being guided by the experience of the team. Strong agreement

Cf argument of recommendation 2.3.1.4

2.3.3 In patients requiring systemic anticoagulation:
2.3.3.1 Systemic anticoagulation using heparin should probably be preferred to other anticoagulants. (Expert opinion) Strong agreement
2.3.3.2 In patients with suspected or proven heparin-induced thrombocytopenia, in addition to cessation of heparin treatment, it is possible to use regional citrate anticoagulation as a complement to the anticoagulation of heparin-induced thrombocytopenia. (Expert opinion) Strong agreement

If there is repeated thrombosis during effective heparin anticoagulation, despite the usual precautions (sufficient blood flow, filtration fraction <20 %, no catheter dysfunction), heparin-induced thrombocytopenia should be suspected and tested for. Regional citrate anticoagulation is certainly the best option.

### Area 2.4: Reverse osmosis water

2.4.1 A quality program should be set up to ensure that the reverse osmosis water meets regulatory standards. (Expert opinion) Strong agreement

The quality of the water used for intermittent RRT techniques in intensive care should comply with the circulars and standards defined in the European pharmacopeia in force for chronic hemodialysis. Regular checks of management of water quality are the responsibility of the pharmacist in association with the intensivist. Water quality control should be guaranteed permanently by setting up a suitable and effective quality assurance system by following the circulars (DGS/DH/AFSSAPS 2000-337, GGS/DH/AFSSAPS 2007-52) and standards (NF S93-310:2004 and NF S93-315: 2008) in force.

## Area 3: Practical aspects

### Area 3.1: Choice of the method

3.1.1 Continuous and intermittent RRT techniques can be used equally, taking into account their availability and the experience of the team. Strong agreement
3.1.2 Diffusive and convective RRT techniques can be used equally, taking into account their availability and the experience of the team. Strong agreement

Several randomized studies and meta-analyses have examined the safety and outcome of patients treated by intermittent hemodialysis and hemofiltration [105–114]. Their data suggest that none of these techniques has an advantage over the others in terms of survival. Several of these studies included hemodynamically unstable patients [108, 109, 111, 113], and one study excluded them [107]. The rate of hemodynamic instability either did not differ significantly [106, 108, 113] or was not in favor of intermittent hemodialysis [105, 112]. Most studies, however, provide limited information on these criteria, and it was only a secondary criterion in most studies. The dialysis modalities, prescribed doses of dialysis, time before initiation and criteria of hemodynamic instability varied greatly between the studies, making their comparison difficult.

The data on the recovery of renal function after RRT are discordant [115, 116]. Observational studies suggest an increased risk of persistent renal failure in patients initially treated by intermittent hemodialysis, whereas data from randomized studies indicate no difference according to technique [107–109, 111, 112, 116]. A recent meta-analysis indicated an increased risk of intermittent techniques [116]. The discordance between the data from observational studies (odds ratio 1.99; 95 % CI 1.53–2.59) and from randomized studies (odds ratio 1.15; 95 % CI 0.78–1.68) prevents the drawing of any definitive conclusion.

The speed of correction of metabolic anomalies is greater with intermittent hemodialysis, but the advantage of intermittent hemodialysis over continuous techniques has not been specifically assessed. There are no literature data indicating a superiority or inferiority of diffusive versus convective modalities.

3.1.3 In patients with brain damage and a risk of intracranial hypertension, continuous or sustained low-efficiency dialysis (SLED) should probably be preferred. (Expert opinion) Strong agreement

Intermittent techniques, unlike continuous techniques, can induce osmotic variations [117], which lead to cerebral edema (dialysis disequilibrium syndrome) [117, 118]. A single observational study indicates that intracranial pressure increases in cranial trauma patients during intermittent hemodialysis [119]. However, no study has compared the neurological morbidity of the two techniques in patients with brain damage.

### Area 3.2: Peritoneal dialysis

3.2.1 In children and neonates, peritoneal dialysis is possible, in particular postoperatively after heart surgery, with a view to salt and water depletion, because of its ease of use. Poor agreement
3.2.2 In children and neonates, peritoneal dialysis is possible for acute renal failure when there is no criterion for emergency dialysis. Poor agreement

Peritoneal dialysis (PD) is an RRT technique that is still much used in pediatric intensive care in both emerging countries and the industrialized world. The main indications reported in the literature are salt and water depletion in the postoperative period after heart surgery and the treatment of isolated acute renal failure. Several studies confirm the efficacy of PD in these indications [120–130]. The time needed to achieve effective dialysis with PD means that it cannot be used when there is an emergency indication. The principal criterion for choosing PD over another technique is that it does not require vascular access for children at risk of terminal chronic renal insufficiency. Three studies of low methodological quality (nonrandomized) compared PD with another RRT technique in a postoperative setting [131] or in different types of renal failure [132, 133]. Two of these studies [132, 133] found an identical mortality, while the third [134] showed excess mortality in a hemodialysis group, but this study suffered from bias in the distribution of the patients by disease severity. One study [132] found that caloric intake was greater in continuous arteriovenous hemofiltration and in continuous veno-venous hemofiltration than in PD. One study [132] showed an association between use of PD and vasopressor treatment. In the indications cited above, it is not possible to conclude that one or other technique is superior to another. In all cases, the choice of a PD technique means that the care team must be completely familiar with the procedures, complications and monitoring.

3.2.3 In adults, peritoneal dialysis should probably not be used first-line. Strong agreement

There are few comparative studies for assessment of the role of PD in the management of acute renal failure requiring RRT in adults. The three studies available are contradictory regarding mortality [134–136]. In contrast, the time needed to achieve satisfactory metabolic control and adequate volume control means that PD is unsuited to life-threatening situations. While PD is useful in certain parts of the world where use of other techniques is difficult, it cannot be recommended when these other methods are available.

### Area 3.3: Dialysis dose

3.3.1 In intermittent RRT the minimum delivered dose of dialysis should probably be (1) three sessions per week of at least 4 h with a blood flow >200 mL/min and a dialysate flow >500 mL/min, or (2) a Kt/V index >3.9 per week, or (3) maintenance of a predialysis urea concentration of 20–25 mmol/L. Strong agreement

Three randomized studies evaluated the impact of the dialysis dose in intermittent RRT in intensive care patients [32, 137, 138]. The first two used the Kt/V index to measure and compare the doses of dialysis delivered [32, 138]. The single-center study of Shiffl et al. compared 3.5-h sessions delivered daily or every 2 days in 160 patients [138]. The weekly Kt/V was 3.0 ± 0.6 in the conventional renal support group versus 5.8 ± 0.4 in the intensive dialysis group. The authors observed a significant 18 % (95 % CI 33 %/4 %) reduction in mortality. Two biases can, however, explain the excess mortality in the conventional group: (1) the delivered dose of dialysis was particularly low, below that required in chronic dialysis patients, (2) the management of the water balance by short sessions every two days required ultrafiltration flow rates that were much higher and poorly tolerated. A second, multicenter study performed in the USA by the VA/NIH in 1124 patients compared two groups treated with 4-h sessions three or six times a week [32]. The average daily Kt/V was 3.93 in the conventional group and 7.1 in the intensive dialysis group. On average, the plasma urea concentration before the sessions was 25 ± 12 mmol/L in the conventional group and 16 ± 9 mmol/L in the intensive dialysis group. Mortality did not differ between the two groups (35.2 % conventional versus 38.2 % intensive). In the third study, prolonged daily sessions of low-efficiency hemodialysis were used in 156 patients to maintain plasma urea at 20–25 mmol/L or <15 mmol/L [137]. The patients received 8 h of treatment/day on average for a mean urea concentration of 19.1 ± 6.8 mmol/L in the conventional group versus 18 h of treatment per day for a urea concentration of 11.4 ± 4.1 mmol/L in the intensive dialysis group. Mortality at day 14 was 29 % in the two groups, which limited the power of this study, the expected mortality of which was 60 %. When considered in a meta-analysis, these studies do not show that a higher dialysis dose was superior in terms of mortality or dependence on chronic dialysis. It is not, however, possible to conclude that the dialysis dose has no impact on the prognosis of intensive care patients, but rather that there is a minimum dose, used in the control arm of these three studies, above which increase provides no further benefit. It is not, however, possible to define a single minimum dose because in these studies the dose and the delivery modalities (clearance, time, frequency) were not identical. Current data do not allow elimination of a possible impact of the delivered dose of dialysis on the prognosis in certain subgroups of patients, such as those with acute renal failure of intermediate severity [139].

3.3.2 In continuous RRT, the minimum delivered dose of dialysis should probably be 20–25 mL/kg/h of effluent, by filtration and/or diffusion. Strong agreement

In continuous RRT, dialysis dose is the total volume of effluent, i.e., the sum of the ultrafiltration volume obtained by convection and the volume of dialysate obtained by diffusion. Single-center and old studies suggest that an increase in dialysis dose in continuous RRT could increase patient survival. Ronco and colleagues reported greater survival of patients receiving 35 mL/kg/h of filtration with reinjection postdilution in comparison with patients receiving 25 mL/kg/h (57 versus 41 %, p 0.0007) [140]. Saudan and colleagues included 206 patients in a randomized trial comparing hemofiltration with a total effluent flow rate of 25 mL/kg/h with hemodiafiltration where a 15 mL/kg/h dialysate flow rate was added to ultrafiltration of 25 mL/kg/h, i.e., a total effluent flow rate of 35 mL/kg/h [141]. Survival at day 90 was significantly higher in the hemodiafiltration group (59 versus 34 %, p = 0.0005).

Two larger randomized, multicenter studies were subsequently designed to confirm or deny the advantages of using a high dialysis dose in continuous RRT. The first was a VA/NIH randomized study of 1124 intensive care patients with acute renal failure, 615 of whom were treated by predilution hemodiafiltration (with a ratio of dialysate to replacement fluid of 1:1) that was intensive (35 mL/kg/h) or conventional (20 mL/kg/h) [142]. Mortality at day 60 was similar in the two groups. The patients receiving intensive treatment had more complications, essentially metabolic disorders. The second study was the Randomized Evaluation of Normal versus Augmented Level (RENAL) Replacement Therapy Study, in which 1508 patients were treated by postdilution hemodiafiltration with a ratio of dialysate to replacement fluid of 1:1. The patients were randomized to intensive (40 mL/kg/h) or conventional (25 mL/kg/h) treatment [143]. Survival at day 90 and recovery of renal function did not differ between the two groups. There were more complications in the patients in the intensive dialysis group. A meta-analysis of seven trials comparing a conventional dialysis dose with an intensive dose in continuous RRT found no difference in the prognosis of the patients (odds ratio 0.87, CI 0.71–1.06).

The VA/NIH and RENAL studies show that increasing the dose of continuous RRT to above 25 mL/kg/h of total effluent is of no benefit in intensive care patients treated for acute renal failure.

3.3.3 The delivered dose of dialysis should be adapted to the patient’s needs in terms of control of metabolism, electrolyte balance and acid–base equilibrium. The onset of hypokalemia and/or hypophosphatemia must be prevented. The dosage of drugs removed by RRT should be adapted to the dose delivered. (Expert opinion) Strong agreement

In an intensive care patient, the dialysis dose should not be defined only by the quantity of urea removed. The aims of RRT are maintenance of water-electrolyte balance, acid–base equilibrium and nutritional balance. The heterogeneity of the patients included in randomized trials and their selection using inclusion criteria prevents recommendation of a conventional dialysis dose adapted to all patients. In addition, the therapeutic aims of RRT have to be adjusted in light of the patient’s progress. An increase of the dialysis dose is required in certain clinical circumstances, such as life-threatening hyperkalemia, severe metabolic acidosis (pH < 7.20), and tumor lysis syndrome [144]. On prescription of a session of RRT, it should be borne in mind that the delivered dose of dialysis is in general below the prescribed dose. The main technical factors that decrease the intensity of RRT are treatment interruption (alarms, moving of the patient), loss of membrane efficiency (clogging and coagulation) and dysfunction of the vascular access route.

Whatever the method, intensification of RRT frequently generates metabolic disorders like hypokalemia and hypophosphatemia, with unfavorable clinical consequences [32, 145, 146]. To prevent underdosage, dosages of drugs, particularly anti-infective agents, should be adapted to the intensity of the RRT [147, 148].

3.3.4 In intermittent RRT, the length and/or frequency of sessions should probably be increased in cases of hypercatabolism and/or severe metabolic disorder and/or an indication for salt and water depletion. (Expert opinion) Strong agreement

The heterogeneity and mild renal failure of patients included in studies comparing different intensities of dialysis delivered in intermittent RRT prevent determination of the minimum dose to be delivered to patients with more severe disease. It should be remembered that in the VA/NIH study the patients treated by intermittent RRT had no hemodynamic impairment [142]. In these patients, the urea production rate, the accumulation of fluid that increases the volume of distribution of the urea and the severity of the metabolic disorders probably necessitate a higher dialysis dose.

Increase in the length or frequency of the sessions depends on the clinical objective. It is clearly established that an increase in session length from 4 h to 6–8 h will raise the efficiency of removal of a solute with a high volume of distribution like urea [149]. Movement of intracellular urea towards the plasma by the generation of a diffusion gradient is time-dependent. The heterogeneity of tissue perfusion results in a mismatch between the stock of urea and the perfusion rate. Frequent, short high-efficiency dialysis sessions (high flow of blood and dialysate) effectively remove solute with a low volume of distribution, i.e., distributed mainly in plasma [150]. Lastly, if the aim of RRT is to control sodium and water balance by depletion, the use of daily sessions seems more effective and better tolerated. In the VA/NIH study, the use of sessions 6 days/7 versus 3 days/7 resulted in a blood volume depletion above 2–3 L/week [32]. In certain studies, it was observed that control of sodium and water balance was facilitated by continuous RRT [151, 152]. This suggests that intermittent techniques should be used daily when large depletion is indicated.

3.3.5 In continuous RRT, the dialysis dose should not be increased for sepsis alone. Strong agreement

Pro- and anti-inflammatory mediators were found in the ultrafiltrate of patients with sepsis treated by continuous hemofiltration [153]. Based on the assumption that hemofiltration nonspecifically reduces the concentration peaks of these mediators and thus has an immunomodulatory effect, this technique was soon put forward as an adjuvant treatment of septic shock. Animal studies gave encouraging results, with a decrease in catecholamine doses in animals treated by high-volume hemofiltration (>50 mL/kg/h). The first clinical experience in human subjects was reported by Honoré and colleagues in 20 patients with refractory septic shock. Using a very big ultrafiltration dose (6 L/h) for 4 h followed by a conventional dose [154], they noted improved hemodynamics, metabolic equilibrium, and survival at day 28 (compared with predicted mortality), when high-volume hemofiltration was initiated early. Several authors have unsuccessfully attempted to show that high-volume hemofiltration confers improved survival in septic shock [155–157]. Finally, Borthwick and colleagues [158] attempted to perform a meta-analysis of the use of high-volume hemofiltration in sepsis using only three randomized studies including a total of 64 patients. Because of the heterogeneity of these three studies, their endpoints, and their small populations, the analysis proved impossible.

Various prospective randomized studies assessing the effect of higher doses of dialysis on mortality have also failed to find a significant effect, even by analysis in the subgroup of patients with septic shock [140, 142, 149–152]. In a meta-analysis of 9 randomized trials comparing two intensities of dialysis in 1786 patients, a specific analysis was done to assess the impact of dialysis dose in the subgroup of septic shock patients. Intensification of the dialysis dose was not associated with reduced mortality (odds ratio 1.02, CI 95 % 0.85–1.23).

Adverse effects associated with the use of high-flow ultrafiltration could counterbalance the modulating effect of this treatment. High-volume hemofiltration may lead to major metabolic disorders (in particular, hypophosphatemia and hypokalemia), deep hypothermia, increased clearance of drugs (particularly antibiotics), and deficiency in micronutrients such as selenium.

Currently available data therefore suggest that sepsis in a patient with renal failure does indicate increase of dialysis dose.

### Area 3.4: Adjustments

3.4.1 It does not seem necessary to use heparin when rinsing RRT circuits. (Expert opinion) Poor agreement

Although there is no corresponding study, it is generally agreed that the circuit should be rinsed with isotonic saline solution and that arterial and venous lines should be connected simultaneously to avoid volume depletion induced by rinsing. Heparinization of the rinsing fluid is debated because no study has tested its usefulness. In hemodialysis, it is not necessary to add heparin when rinsing the circuit. In hemofiltration, usual practice is to use 5000 IU of heparin in the second liter of fluid when rinsing the circuit so as to extend filter lifespan and increase adsorption capacity (no study). This approach, however, has not been validated by scientific evaluation.

3.4.2 Nutritional intake of RRT patients should not be reduced. (Expert opinion) Strong agreement
3.4.3 In hemofiltration post-dilution, blood flow should be adjusted to keep the filtration fraction below 25 %. (Expert opinion) Strong agreement

The estimated minimum blood flow is 150 mL/min. The filtration fraction should be kept below 25 % when there is unfractionated heparin anticoagulation (no study available). However, the best indicator of viscosity within the filter is the hematocrit, which should remain below 40 % (no study available). Measurement of the hematocrit in the filter enables much better determination of the appropriate filtration fraction, particularly when predilution is used. The filtration fraction should be corrected according to the amount of predilution used. Routine use of the hematocrit in the filter obviates the need for these tedious calculations. The use of citrate, currently based on more effective anticoagulation, optimizes therapy while tolerating higher (up to 27 %) filtration fractions (no studies available).

3.4.4 In intermittent hemodialysis of duration <6 h, the blood flow rate in dialysis should be between 200 and 300 mL/min and the dialysate flow rate ≥500 mL/min for most patients. (Expert opinion) Strong agreement
3.4.5 In children, in intermittent hemodialysis of duration <6 h, the blood flow rate should start at 3 mL/kg/min and increase to 5 mL/kg/min for the following sessions, and the dialysate flow rate should be at least 300 mL/min up to twice the blood flow rate in mL/min. (Expert opinion) Strong agreement

Recommendations concerning session length and flow rates in intermittent hemodialysis are based on two main objectives: (1) delivery of the dialysis dose recommended, (2) satisfactory hemodynamic tolerance. The recommendations are therefore principally based on arguments related to the dose of dialysis (see recommendation 3.3.1). So these recommendations apply to most patients. In certain clinical situations, it may be necessary to make adjustments essentially to prevent dialysis disequilibrium syndrome, which may occur on initiation of RRT in patients with a high urea concentration. The aim is to avoid large variations in osmolality so as to prevent cerebral edema. There are no clearly defined settings in this situation, but a sharp drop in plasma urea concentration should be avoided. The length of the session and the blood flow, and even the dialysate flow rate, should therefore be reduced. Note that use of a sodium-enriched dialysate, as recommended when there is hemodynamic instability (see recommendation 3.4.8), probably prevents this risk.

3.4.6 In sustained low-efficiency dialysis, low blood and dialysate flow rates should be used. (Expert opinion) Strong agreement

Intermittent sustained low-efficiency hemodialysis (SLED) has been proposed as a hybrid of intermittent short-term high-efficiency hemodialysis and continuous low-efficiency methods. Using a classic hemodialysis machine for a duration of 8–12 h, SLED enables slower dialysis and management of sodium and water balance over a longer period, while remaining intermittent. The expected effects are improved hemodynamic tolerance, reduced risk of dialysis disequilibrium syndrome, and increased dose delivered by plasma refilling (equilibration of urea concentrations between plasma and interstitial tissue), which is made possible by the prolonged session. This modality has been little evaluated and numerous protocols have been proposed, all based on a decrease in blood and dialysate flow rates, without it being possible to infer recommendations for clinical practice [159–161].

3.4.7 The arterial and venous lines should be connected simultaneously to avoid volume depletion. (Expert opinion) Strong agreement
3.4.8 In intermittent hemodialysis, lowering of dialysate temperature should probably be recommended, to improve hemodynamic tolerance. Strong agreement
3.4.9 In intermittent hemodialysis, sodium concentration in the dialysate (conductivity) should probably be increased to >145 mmol/L, to improve hemodynamic tolerance or when the urea concentration is very high. Strong agreement
3.4.10 In intermittent hemodialysis, a bicarbonate buffer should probably be used. Strong agreement

Recommendations 3.4.6–3.4.10 concern the conditions recommended for optimization of hemodynamic tolerance in intermittent hemodialysis. This is important as intradialytic hypotension during intermittent hemodialysis sessions can generate ischemia–reperfusion episodes, which maintain or worsen tubular necrosis. The influence of thermal balance, dialysate composition, and sodium and water balance has been demonstrated above all in chronic dialysis, in improving tolerance of blood pressure changes in frail patients. Use of a moderately cooled dialysate limits warming induced by RRT which is accompanied by a decrease in vasomotor tone [162]. For the dialysate, the choice of buffer and the sodium conductivity are important. Acetate buffer, which induces vasoplegia and contractile dysfunction [163], has been replaced by bicarbonate. High sodium conductivity results in increased plasma sodium and so limits the rapid drop in osmolality, notably at the start of the session. This helps improve hemodynamic tolerance [164]. Few studies have investigated the influence of these guidelines in intensive care patients undergoing dialysis because of acute renal failure. One study in intensive care that assessed the effect of these guidelines (bicarbonate buffer, dialysate enriched in sodium and moderately cooled, isovolemic connection and rational ultrafiltration) showed a clear improvement in hemodynamic tolerance [165]. Application of these guidelines in a prospective randomized study showed that hemodynamic tolerance of the resulting intermittent hemodialysis was comparable with that of continuous RRT, which is reputed to be less likely to result in hemodynamic instability [108].

## Area 4: Safety

### Area 4.1: Procedures, training

4.1.1 RRT should be set up using an in-house procedure including at least specific prescription and monitoring, description of the technical procedures and required hygiene precautions and disinfection of monitors/hemodialysis machines. (Expert opinion) Strong agreement
4.1.2 The medical and paramedical teams should be trained in accord with professional standards, so they acquire the required skills in using RRT monitors/hemodialysis machines, in the prevention and treatment of complications of the different techniques and in the traceability of events and hygiene procedures. (Expert opinion) Strong agreement

RRT is a highly technical procedure involving a chain of interventions subject to the risk of human error and/or equipment failure. In 2008 the SRLF and the SFAR drew up recommendations to ensure the safety of RRT [166].

RRT requires complex equipment and precise settings. Nurses play the main role in RRT [167]. When the technique used is hemodialysis, adjustments have a direct impact on tolerance and the use of good practice procedures significantly reduces complications [165, 168, 169]. Although little evaluated, there are many arguments in favor of management of RRT techniques based on standard procedures and for a training program specific to these techniques.

Definition of healthcare procedures is a recognized way of improving intensive care practices [170, 171]. Understanding of the content and aims of these procedures is, however, essential if they are to be applied correctly. Expert recommendations and experience indicate that management of RRT sessions by trained personnel using standardized procedures is necessary [167, 168, 172–176]. In a recent survey of renal failure management in 188 intensive care departments in the United Kingdom, 73 % of the departments reported that they had a standardized procedure for RRT [177]. There are numerous infectious risks associated with RRT. The French Society for Hospital Hygiene has drawn up recommendations stipulating that good hygiene practices in performing RRT must be applied in accordance with specific procedures established by the healthcare teams and approved by the institution and that the personnel must be trained in these procedures [178]. In addition to standard precautions for the prevention of bacterial and viral transmission, management of the disinfection of the hemodialysis machine should also be included [178].

Training of intensive care personnel in RRT is made difficult by the low frequency of use of this technique. A study carried out between 1997 and 2007 in 108 intensive care units in France and the USA showed that one quarter of the units treated fewer than 10 patients/year [179]. Frequent staff turnover, the predominance of young qualified nurses and the technique-dependent specificity of the equipment used are additional hindrances to acquisition of the required skills [168]. Healthcare teams should ideally have access to structured training in RRT, with assessment of learning.

### 4.2 Dialysis catheter management

4.2.1 Use of a dialysis catheter should be reserved for RRT. (Expert opinion) Strong agreement

Because of its specificity (diameter, length, locking solution) and indication for placement (use of RRT), the dialysis catheter in intensive care should not be used for purposes other than RRT, so as to minimize the risk of complications associated with dialysis catheters: thrombosis, dysfunction, and infection.

4.2.2 RRT catheters should be handled according to the recommendations applicable to central venous catheters. (Expert opinion) Strong agreement

Most data on management of temporary dialysis catheters come from studies on indwelling dialysis catheters in patients with end-stage renal failure or from studies on central venous catheters in intensive care. Although the profile of intensive care patients differs from that of chronic renal failure patients, and although dialysis catheters are different from central venous catheters in terms of handling, management, and complications (dysfunction, thrombosis, infection), the data suggest that dialysis catheters should be handled according to the recommendations applicable to central venous catheters [34, 37, 38, 180]

4.2.3 Failure to attain or maintain a blood flow rate necessary and sufficient to deliver an adequate dose of treatment should probably be considered as a criterion of RRT catheter dysfunction. (Expert opinion) Strong agreement

There is no consensual definition of dialysis catheter dysfunction in intensive care patients. In patients undergoing chronic hemodialysis, catheter dysfunction is conventionally defined using hemodynamic criteria. In intensive care, catheter dysfunction should be considered when it is impossible to aspirate the blood in at least one of the lines or to deliver a catheter blood flow rate >150 mL/min [181]. Dysfunction can also be defined by the inability to administer the prescribed dialysis dose despite lowering prepump arterial pressure to −250 mmHg or raising venous pressure to 250 mmHg [43].

4.2.4 Hypovolemia should be eliminated when there is catheter dysfunction. (Expert opinion) Strong agreement
4.2.5 Thrombosis should be eliminated when there is catheter dysfunction unrelated to hypovolemia. (Expert opinion) Strong agreement

Catheter dysfunction generally occurs early (<10 days) [43, 181] and is therefore associated with positioning problems or blood flow below the rate required for RRT. There is a greater risk of femoral catheter dysfunction if its distal end does not reach the inferior vena cava [43]. Likewise, there is a greater risk of internal jugular catheter dysfunction if its distal end does not reach the right atrium [182]. Late-occurring catheter dysfunction is principally related to catheter thrombosis, partial or complete obstruction of the catheter, or thrombosis outside the catheter in the cannulated vein [183].

4.2.6 In intermittent hemodialysis in adults, the RRT catheter should be changed as soon as possible if the lines have to be swapped, in the absence of hypovolemia. (Expert opinion) Strong agreement

If the lines are swapped, recirculation may reduce the RRT dose administered during techniques that use high blood flow. If the aim is to deliver an adapted dose, only a change of catheter is indicated, once blood volume is restored.

4.2.7 It is not possible to recommend one type of locking solution rather than another (saline, heparin, citrate). (Expert opinion) Poor agreement

RRT catheter dysfunction is a frequent event that can lead to premature catheter replacement. Data on the influence of the locking solution (heparin, NaCl, citrate, ethanol) on dysfunction are scarce and essentially concern end-stage renal failure patients or venous access at the jugular site [184–186]. Data from the study group Cathedia show no difference in catheter dysfunction between the jugular and femoral sites [43], a conclusion backed up by a recent small, single-center, randomized, open study of the effect of citrate locking solution on these 2 sites commonly used in intensive care (similar proportion in the 2 groups) [187]. The result of the principal outcome was in favor of the use of the citrate locking solution. There is also a “signal” concerning catheter infections, which did not differ in number between the two groups, but for which the time to onset was longer in the citrate locking solution group. This result could be due to bacteriostatic effects of the citrate, the role of which in these infections has yet to be investigated in a study of suitable size. Nonetheless, heparin locking solutions are also widely used around the world, and a large-scale comparison of citrate locking solution, heparin locking solution, and saline locking solution is essential if the value of citrate locking solution is to be assessed in a cost-benefit analysis.

### 4.3 Extracorporeal circuit set-up

4.3.1 Connecting the circuit:
  4.3.1.1 Before connecting the circuit, the patency of the vascular access should be checked. (Expert opinion) Strong agreement
  4.3.1.2 Two people are needed to set up the catheter lines. (Expert opinion) Strong agreement
  4.3.1.3 Optimal rinsing of the extracorporeal circuit is needed to minimize the amount of air, so as to reduce the risk of coagulation and of gas microembolism. (Expert opinion) Strong agreement
  4.3.1.4 Connections between the vascular access and the extracorporeal circuit should be kept visible during dialysis to reduce the risk of inadvertent disconnection. (Expert opinion) Strong agreement
  4.3.1.5 Patient agitation should be prevented to avoid inadvertent disconnection. (Expert opinion) Strong agreement
  4.3.1.6 Blood flow in the extracorporeal circuit should be increased progressively to check that the vascular access is patent and the circuit airtight. (Expert opinion) Strong agreement
  4.3.1.7 In hemofiltration, convection should be started when the target blood flow is attained, so as to avoid excess hemoconcentration in the filter. (Expert opinion) Strong agreement
4.3.2 During the session
  4.3.2.1 Circuit pressures (arterial, venous, transmembrane) and pressure drop should be monitored closely. (Expert opinion) Strong agreement
  4.3.2.2 The lines of the extracorporeal circuit should be fastened to avoid kinking and resultant stoppage of the blood pump. (Expert opinion) Strong agreement
  4.3.2.3 The blood pump flow rate should be reduced and convection interrupted when moving the patient. (Expert opinion) Poor agreement
  4.3.2.4 Aseptic technique should be followed and introduction of air avoided when sampling blood in the extracorporeal circuit. (Expert opinion) Strong agreement
  4.3.2.5 The blood level in the bubble trap should be kept high to reduce the incidence of gas microembolism. (Expert opinion) Strong agreement
4.3.3 Disconnecting the circuit
  4.3.3.1 Saline should be used to return blood in the extracorporeal circuit to the patient. (Expert opinion) Strong agreement
  4.3.3.2 On disconnection, the patient should be placed in the supine position to reduce the risk of gas embolism. (Expert opinion) Poor agreement
  4.3.3.3 In infants weighing under 15 kg, the return blood flow should probably be less than or equal to 2 mL/kg/min. (Expert opinion) Poor agreement

These recommendations concerning practical management of RRT sessions are all expert opinions based on the rules of good practice. No scientific publication has evaluated their impact. However, it seemed necessary for clinical practice to provide inexperienced teams with solid guidance on how to improve the implementation of these techniques.
